# Dual inhibitory potential of N-methylcytisine against GSK-3β and AChE: implications for Alzheimer’s disease treatment

**DOI:** 10.1515/biol-2025-1363

**Published:** 2026-08-17

**Authors:** Shirin Tarbiat, Nigar Kantarci-Carsibasi

**Affiliations:** Department of Molecular Biology and Genetics, Uskudar University, 34662 Istanbul, Türkiye; Department of Chemical Engineering, Uskudar University, 34662 Istanbul, Türkiye

**Keywords:** N-methylcytisine, GSK-3β, AChE, molecular dynamics, DFT calculations

## Abstract

Alzheimer’s disease (AD) is a progressive neurodegenerative disease characterized by memory loss and irreversible cognitive decline. Glycogen synthase kinase 3β (GSK-3β) is significant in tau hyperphosphorylation and neurodegeneration. The cholinergic hypothesis of AD links cognitive impairment to reduced synaptic acetylcholine (ACh). Increased acetylcholinesterase (AChE) activity exacerbates this issue. To search for a potential dual GSK-3β/AChE inhibitor, we focused on N-methylcytisine. This natural cytisine-derived alkaloid has not been studied for its various biological effects on the prevention of AD. The *in vitro* results indicated that N-methylcytisine displayed promising activity against GSK-3β and AChE with IC_50_ values of 11 and 22.7 µM, respectively. GSK-3β kinetic study according to the varying substrate or ATP concentrations at different N-methylcytisine levels revealed mixed-type inhibition. The results of the integrated in silico workflow indicate that N-methylcytisine exhibited favorable binding to AChE (docking score −9.6 kcal/mol; MM-GBSA −70.2 kcal/mol), comparable to the reference inhibitor galantamine (−10.8 kcal/mol; −70.6 kcal/mol). In contrast, its interaction with GSK-3β was more moderate (−5.3 kcal/mol; −50.3 kcal/mol) relative to staurosporine (−8.5 kcal/mol; −82.6 kcal/mol), consistent with its smaller scaffold. Overall, the results support a flexible, mixed-type interaction profile and highlight N-methylcytisine as a promising dual-acting candidate for Alzheimer’s disease.

## 1 Introduction

Alzheimer’s Disease (AD) is a progressive, age-related neurodegenerative disease characterized as the most common form of dementia in elderly individuals. Its features include behavioral disturbances and progressive, untreatable cognitive and functional deterioration. Although the molecular mechanisms of AD pathogenesis are not well characterized, oxidative stress, chronic neuroinflammation, aggregation of amyloid-β (Aβ), and hyperphosphorylated tau are considered central processes leading to neuronal loss and dysfunction in the development of AD. The major neuropathological lesions of AD are the extracellular Aβ plaques and the intracellular neurofibrillary tangles (NFTs) that contain hyperphosphorylated tau, and they play a core role in the disease pathology. Aβ peptides are produced by the amyloid precursor protein (APP) by consecutive cleavage of β- and γ-secretase. Their deposition interferes with synaptic signaling, elicits glial responses, increases vascular permeability of the blood-brain barrier (BBB), and induces neuronal apoptosis [1].

Hyperphosphorylation of tau associates with changes in its function beyond microtubule destabilization and neurofibrillary tangle formation. Hyperphosphorylation of tau is linked to alterations in its function. Among the kinases implicated in tau dysregulation, glycogen synthase kinase-3β (GSK-3β) is considered a principal pathological driver of tau hyperphosphorylation in AD. Glycogen synthase kinase-3 (GSK-3) has two isoforms (GSK-3α and GSK-3β) with 98 % catalytic similarity. Nevertheless, several studies show that GSK-3β is the isoform that is most dysregulated in tau hyperphosphorylation and neurodegeneration. Highly active GSK-3β not only phosphorylates tau at pathological sites but also enhances β-secretase (BACE1) expression, promotes APP cleavage, and accelerates Aβ deposition [2].

The irreversible, non-ATP-competitive GSK-3β inhibitor tideglusib, advanced to Phase II clinical trials. Despite good tolerability, clinical results did not show a significant improvement in cognitive function, while preclinical studies demonstrated neuroprotective and anti-tau effects [3]. These findings underscore the therapeutic relevance of GSK-3β while highlighting the need for more selective and potent inhibitors.

Meanwhile, the cholinergic hypothesis of AD attributes cognitive impairment to low synaptic acetylcholine (ACh) levels and loss of basal forebrain cholinergic neurons. The situation is worsened by increased acetylcholinesterase (AChE) activity. In this respect, there is a need for symptomatic treatments for broader use, as the currently available ones, such as memantine, galantamine, rivastigmine, and donepezil, have only transitory beneficial effects on cognitive function without modifying neurodegeneration. As both AChE and GSK-3β are the regulators of several cascades implicated in Aβ deposition, tau hyperphosphorylation, and synaptic dysfunction, combined inhibition of them may present an alternative multitarget strategy [4].

Natural compounds with combined AChE and GSK-3β inhibitory action have supported this theory. Flavonoids such as quercetin and kaempferol inhibit AChE while attenuating GSK-3β activity [5], whereas alkaloids like berberine and harmine similarly suppress cholinesterase function and GSK-3β-mediated tau phosphorylation [6], 7]. By modifying cholinergic transmission and downregulating GSK-3β signaling, polyphenols such as curcumin and resveratrol have shown multitarget neuroprotective effects [8], 9]. These findings reinforce the therapeutic potential of natural scaffolds for AD treatment.

Cytisine, a naturally occurring quinolizidine alkaloid and partial agonist of nicotinic acetylcholine receptors (nAChRs), is well-known for its role in smoking cessation. Moreover, cytisine and its derivatives exhibit crucial neuroprotective characteristics, including modulation of cholinergic neurotransmission, calcium regulation, cytoprotective, antioxidant, and antidepressant properties. Given the established involvement of cholinergic dysfunction and nAChR impairment in AD, cytisine and its analogues represent promising candidates for neuroprotective drug development [10].

N-methylcytisine, a naturally occurring analogue of cytisine, shares its core quinolizidine structure [11], yet remains underexplored. Considering that impaired cholinergic transmission and hyperactive GSK-3β signaling converge to drive Aβ accumulation, tau hyperphosphorylation, synaptic failure, and cognitive impairment, dual inhibition of AChE and GSK-3β by structurally simple natural molecules such as N-methylcytisine represents an attractive therapeutic strategy. To date, no systematic investigation has assessed its biological potential in this context.

This study provides the first comprehensive evaluation of the *in vitro* inhibitory activity and kinetic profile of N-methylcytisine against AChE and GSK-3β, complemented by an integrated in silico workflow including molecular docking, molecular dynamics simulations, MM-GBSA calculations, DFT-based quantum mechanical calculations, and ADMET profiling to clarify its binding interactions and mechanistic properties. The structural and electronic characteristics of N-methylcytisine were further examined using DFT-based quantum mechanical analyses, offering new insights into its potential as a dual-target neuroprotective agent for Alzheimer’s disease.

## 2 Materials and methods

### 2.1 Materials

The GSK-3β Kinase Enzyme System kit (Cat. No. V1991) was obtained from Promega GmbH (Walldorf, Germany). All other chemicals and reagents, including N-methylcytisine, acetylthiocholine iodide, DTNB (5,5′-dithiobis-(2-nitrobenzoic acid)), and galantamine hydrobromide, were purchased from Sigma-Aldrich (St. Louis, MO, USA) unless otherwise stated.

### 2.2 Acetylcholinesterase (AChE) inhibition assay

The inhibitory effect of N-methylcytisine on acetylcholinesterase (AChE) was assessed using the Ellman method, which measures the amount of thiocholine produced by detecting the chromophore 5-thio-2-nitrobenzoate at 412 nm [12], 13]. The reaction mixture contained 200 μL of crude AChE solution (0.415 U/mL in 0.1 M phosphate buffer, pH 8.0), 100 μL of DTNB solution (3.3 mM in 0.1 M phosphate buffer, pH 7.0, supplemented with 6 mM NaHCO_3_), N-methylcytisine at final concentrations of 10–200 μg/mL, and 500 μL of phosphate buffer (pH 8.0). Following preincubation of the enzyme with N-methylcytisine for 20 min at 25 °C, the reaction was initiated by adding 100 μL of acetylthiocholine iodide (0.1 mM) as substrate. A Shimadzu UV-2600 spectrophotometer was used to measure the change in absorbance at 412 nm in order to track enzymatic activity for 3 min at 25 °C. Crude AChE was produced from freshly dissected brains of mature Wistar rats according to standard techniques. Briefly, brain tissues were homogenized in ice-cold 0.1 M phosphate buffer (pH 7.4) using a glass–Teflon homogenizer and centrifuged at 10,000×*g* for 15 min at 4 °C. The supernatant was collected and used immediately as an enzyme source. Galantamine hydrobromide (0.01–1.0 μg/mL) was included as a positive control inhibitor. All measurements were performed at least in duplicate, and inhibition was expressed relative to the vehicle-treated control.

### 2.3 GSK-3β inhibition assay (ATP-Glo luminescent assay)

The GSK-3β inhibition assay was performed according to previously published protocols [7]. Briefly, a 500 µM stock solution of the test compound, N-methylcytisine, was prepared in double-distilled water and subsequently diluted with the assay working buffer to obtain final concentrations ranging from 3 to 25 µM. A volume of 1 µL of each test concentration was added to the wells of a 384-well low-volume plate. Next, 1 µL of active GSK-3β enzyme (0.1 μg/μL) was added to each well, followed by 1 µL of 1× assay buffer (prepared by diluting the 5× supplemented buffer containing 50 µM DTT and 50 µM ATP). Finally, 2 µL of phosphor-glycogen synthase peptide-2 (GS-2) substrate solution (0.3 μg/μL) was added, bringing the total reaction volume to 5 µL. The reaction mixture was incubated at 25 °C for 60 min, after which 5 µL of ADP-Glo reagent was added and incubated for an additional 40 min at 25 °C. Subsequently, 10 µL of kinase detection reagent was added, and the plate was incubated for a further 30 min at 25 °C. Luminescence was then measured to determine the inhibitory activity of N-methylcytisine against GSK-3β.

### 2.4 Mechanism of GSK-3β inhibition by N-methylcytisine

Jump dilution assay: This assay was performed to assess whether the inhibitor binds reversibly or irreversibly to GSK-3β [7]. This experiment is particularly useful to confirm the reversibility of enzyme inhibition before conducting detailed kinetic analyses. The objective of the assay was to reduce the inhibitor concentration below its IC_50_ value following preincubation. The overall procedure followed the same conditions as the GSK-3β inhibition assay. The enzyme was first preincubated with either a high inhibitor concentration (110 µM, corresponding to 10 × IC_50_ of N-methylcytisine) or with vehicle control (without inhibitor) for 30–60 min under standard assay conditions. Following preincubation, the mixtures were rapidly diluted 50-fold into a reaction buffer containing ATP and substrate but no inhibitor to initiate the phosphorylation reaction.

This dilution step reduced the inhibitor concentration from 110 µM to approximately 2.2 µM, well below its IC_50_ value of 11 µM, while maintaining an active enzyme concentration suitable for measurement. Restoration of enzymatic activity after dilution indicated reversible inhibition, whereas persistent loss of activity suggested irreversible or time-dependent binding. Residual enzyme activity was quantified relative to the vehicle-treated control and expressed as a percentage. Data are presented as mean ± SD from two independent experiments. The kinetic experiments were performed to investigate the inhibitory mechanism of N-methylcytisin on GSK-3β. Lineweaver-Burk plots of enzyme kinetics varying N-methylcytisine concentrations (5, 11, 20 µM) were performed. Initially, the substrate concentration was set at 0.05–0.3 μg/μL, while the ATP concentration remained unchanged. Then, the concentration of ATP was set at 10–50 µM, while the concentration of substrate was kept constant at 0.3 μg/μL.

### 2.5 Geometry optimization by density functional theory (DFT) calculations

Initial conformational sampling of N-methylcytisine was performed using the CSearch module of the Schrödinger Materials Science Suite. employing the OPLS4 force field [14] and low-mode sampling with up to 200 steps. A maximum atomic deviation of 0.5 Å and a 5 kcal/mol energy window were applied, yielding two distinct conformers. The lowest-energy conformer was subjected to geometry optimization using the Jaguar module, Schrödinger’s quantum-mechanics–based optimization engine [15]. Density functional theory (DFT) calculations were performed using the B3LYP-D3 functional with the 6-31G** basis set [16], [17], [18]. SCF accuracy was set to “quick,” with the atomic-overlap method used for initial guess generation. Default maximum SCF iterations and a cap of 100 optimization steps were applied. These calculations provided refined structures and associated electronic properties, including HOMO, LUMO, electrostatic potential maps, electron density distributions, and related descriptors.

### 2.6 Protein preparation

Protein preparation was carried out using the Protein Preparation Wizard in Schrödinger’s Maestro Molecular Modeling Suite v13.1 [19]. The crystal structures of the two target proteins, AChE (PDB ID: 4EY6) and GSK-3β (PDB ID: 4PTC), complexed with their native co-crystallized ligands were retrieved from the Protein Data Bank. Structures were processed by assigning correct bond orders and adding missing hydrogen atoms. Missing loops or side chains were constructed using the Prime module. Because the binding sites were defined around the native ligands, all heteroatoms except the co-crystal ligands were removed. To retain relevant interactions within the active site, crystallographic water molecules within 5 Å of the binding cleft were preserved, and the remaining water molecules were deleted. Protonation states were assigned at pH 7.0 using PROPKA. A restrained minimization was subsequently performed using the OPLS2005 force field [20], applying an RMSD convergence threshold of 0.3 Å.

### 2.7 Ligand preparation

N-methylcytisine (PubChem ID: 670971) and the reference inhibitors Galantamine (PubChem ID: 9651) and Staurosporine (PubChem ID: 44259) were downloaded in 3D SDF format. Because N-methylcytisine is a natural product, the approved natural-source drugs Galantamine and Staurosporine were selected as control molecules for AChE and GSK-3β, respectively. Ligand structures were processed in the LigPrep module of Maestro. Ionization states and potential tautomers were generated with Epik at physiological pH (7.0 ± 2.0). Stereoisomers were enumerated based on the chiral centers present in the 3D structures.

### 2.8 Molecular docking

Molecular docking studies were performed using the Glide module in standard precision (SP) mode [21]. Receptor grids were generated around the binding regions of AChE and GSK-3β, centered on their respective co-crystal ligands. The docking grid encompassed a 20 Å region surrounding the co-crystallized inhibitors (Galantamine for 4EY6, HET ID: GNT; and a carboxamide inhibitor for GSK-3β, HET ID: 2WE). Ligands were treated as fully flexible, and Epik state penalties were incorporated in the scoring function. Docking validation was performed through redocking of the native co-crystal ligands, producing a maximum RMSD of 0.18 Å, confirming that the protocol accurately reproduces the experimental binding pose.

### 2.9 Molecular dynamics (MD) simulations

Molecular dynamics simulations were conducted using the Desmond engine to characterize protein–ligand stability and conformational behavior at atomic resolution. For each protein–ligand system (AChE–galantamine, AChE-N-methylcytisine, GSK-3β–staurosporine, and GSK-3β–N-methylcytisine), three independent 100 ns simulations were performed. A time interval of 1,000 ps was used to record 100 frames per production run, and analyses were performed using averaged trajectories across the entire simulation period. Systems were constructed in an orthorhombic box of 10 × 10 × 10 Å and solvated using the TIP3P water model. Neutralization was achieved by adding 0.15 M NaCl. All simulations were carried out in an NPT ensemble at 300 K and 1 bar using the Nose–Hoover chain thermostat and Martyna–Tobias–Klein barostat [22]. The OPLS2005 force field and RESPA integrator [23] were employed to compute bonded, nonbonded, electrostatic, and van der Waals interactions.

### 2.10 MM-GBSA free energy calculations

Because docking scores alone provide a qualitative estimate of binding affinity, MM-GBSA calculations were performed for a more rigorous assessment of protein–ligand binding free energies. Binding free energies were computed using the Prime MM-GBSA module in Schrödinger. Structures were extracted from the 100 ns MD trajectories at 10 ns intervals, generating 10 frames. MM-GBSA calculations were carried out on every frame, and the reported values represent averages over the 10 frames, reflecting the complete trajectory. The VSGB 2.0 implicit solvation model was used [24]. Minimization was performed with residues within 3 Å of the ligand allowed to remain flexible, using the OPLS2005 force field, consistent with previous steps.

### 2.11 ADMET analysis

N-methylcytisine and control molecules, Galantamine and Staurosporine, are subjected to drug-likeness by checking Lipinski’s rule of five [25] violation. To predict ADMET (absorption, distribution, metabolism, excretion, and toxicity), SwissADME [26] and Pubchem, and ADMETlab 2.0 [27] servers were used.

### 2.12 Statistical analysis

The mean ± standard deviation is used to represent data. Enzyme kinetics were performed using GraphPad Prism software version 9.5.1.

## 3 Results

### 3.1 Inhibitory activity of N-methylcytisine against AChE and GSK-3β

N-methylcytisine exhibited dose-dependent inhibitory effects on both acetylcholinesterase (AChE) and glycogen synthase kinase-3β (GSK-3β). In the Ellman assay, N-methylcytisine reduced AChE catalytic activity with an IC_50_ of 22.7 µM, indicating intermediate cholinesterase inhibition relative to galantamine as a reference compound. In the ADP-Glo™ kinase assay, N-methylcytisine inhibited GSK-3β with an IC_50_ of 11 µM, indicating a moderate inhibitory range in comparison to the reference compound Staurosporine Table 1.

**Table 1:** Inhibitory activity of N-methylcytisine against AChE and GSK-3β.

Compound	AChE IC_50_ (µM)	GSK-3β IC_50_ (µM)
N-methylcytisine	22.7 ± 4^*^ µM	11 ± 2^*^ µM
Galantamine hydrobromide	0.45 ± 0.01	–
Staurosporine	–	0.0804 ± 0.004

Values are expressed as mean ± SD from three independent experiments (*n* = 3). Galantamine hydrobromide and staurosporine were used as reference inhibitors for AChE and GSK-3β, respectively. Statistical comparisons were performed between N-methylcytisine and the corresponding reference inhibitor for each enzyme using Welch’s *t*-test. *P < 0.05 versus the corresponding reference inhibitor.

**Figure 1:**
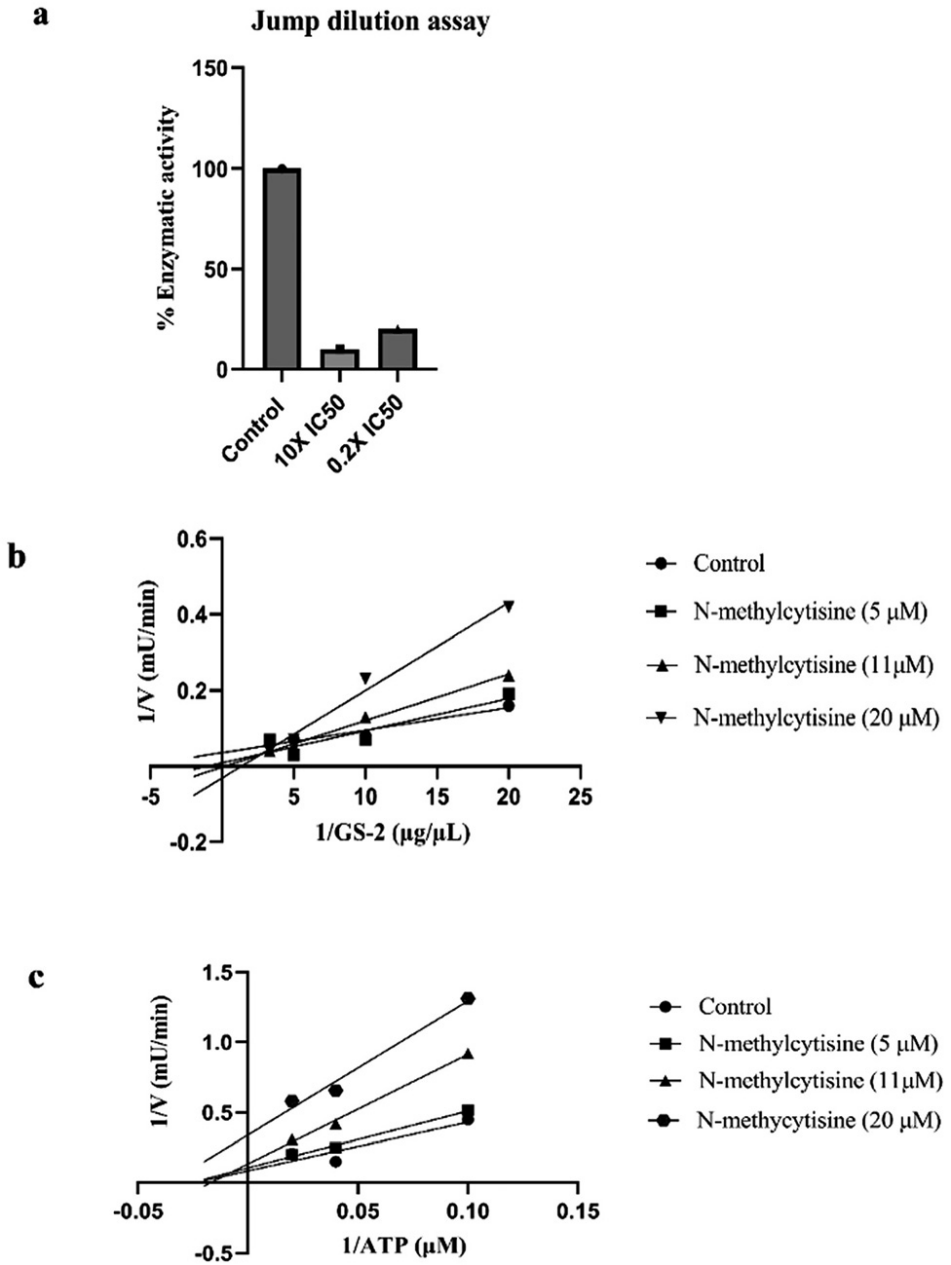
Mechanism of GSK-3β Inhibition by N-methylcytisine. (a) Jump dilution experiment to verify whether the binding between N-methylcytisine and the enzyme is reversible; (b) The enzymatic kinetics of different concentrations of N-methylcytisine at different substrate concentrations; (c) The enzymatic kinetics of different concentrations of compounds at different ATP concentrations.

To determine the mechanism of inhibition of GSK-3β by N-methylcytisine and to further characterize the interactions of the inhibitor with the enzyme, kinetic analyses were performed by varying substrate (GS-2) or ATP concentrations at different N-methylcytisine levels. Lineweaver–Burk plots by nonlinear regression analysis of Graphpad Prism, revealed that the interaction was mixed-type inhibition and demonstrated intersecting lines neither on the *x*-axis nor *y*-axis (Figure 1), consistent with mixed-type inhibition, indicating that N-methylcytisine can interact with both the free enzyme and the enzyme–substrate complex. These results suggest a dual interaction mode involving both the ATP-binding region and potential allosteric or substrate-adjacent binding sites. Staurosporine functioned as the reference control and validated assay performance.

### 3.2 Geometry optimization by density functional theory (DFT) calculations

**Figure 2:**
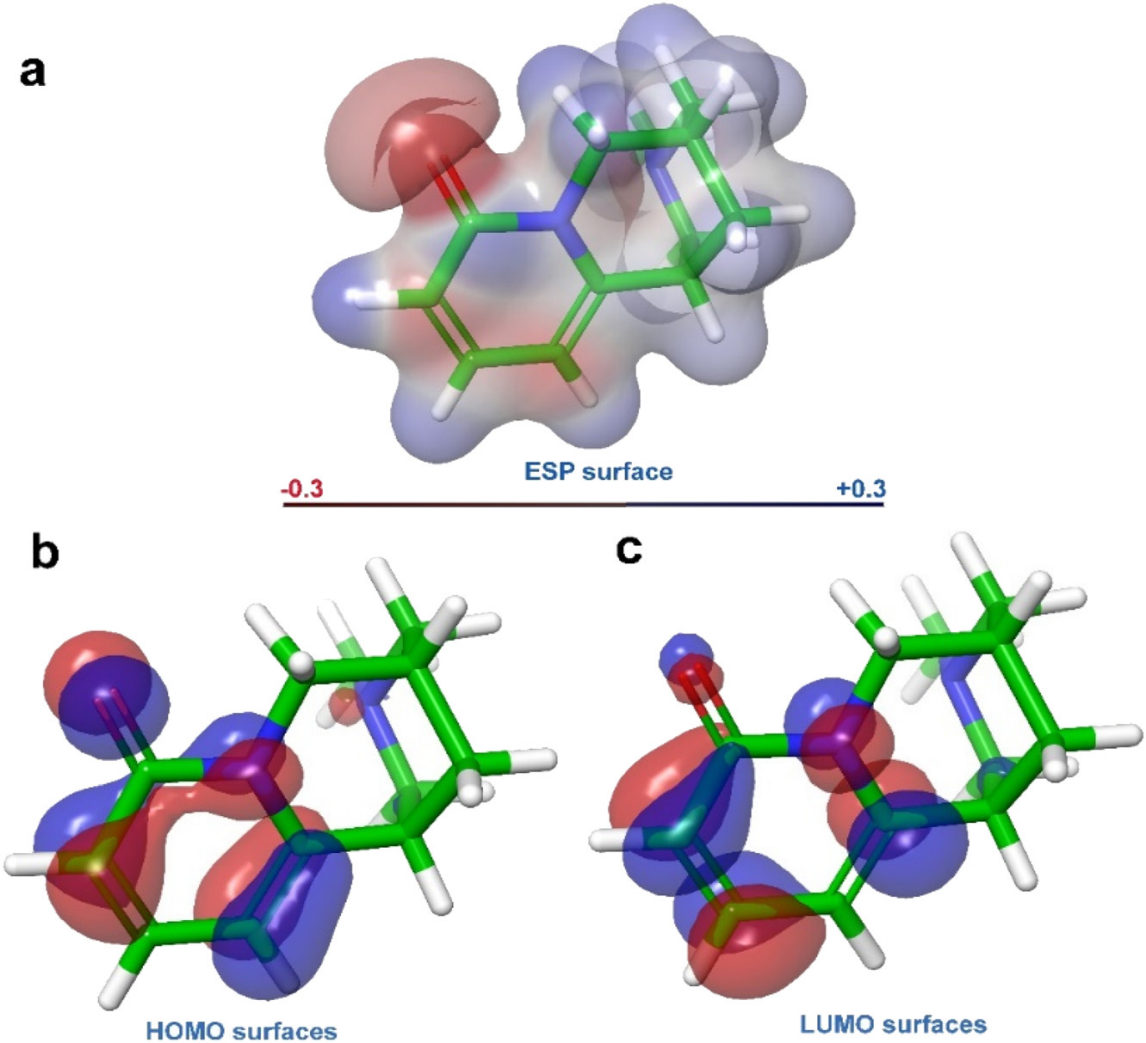
Electrostatic potential and frontier molecular orbital surfaces of N-methylcytisine. (a) ESP isosurface mapped onto the electronic density (isovalue = 0.001 e/bohr³), showing regions of negative potential (red) concentrated around the carbonyl oxygen and regions of positive potential (blue) distributed across the polycyclic scaffold. (b) HOMO distribution showing electron density primarily localized over the aromatic ring system and adjacent nitrogen environment. (c) LUMO distribution highlighting electron-accepting regions centered around the carbonyl moiety and extended π-system. Together, these surfaces illustrate the electronic distribution and potential interaction sites of N-methylcytisine relevant to molecular recognition.

Geometry optimization performed with Jaguar yielded a stable minimum-energy structure for N-methylcytisine, with a gas-phase electronic (SCF) energy of −651.70 Hartree. The electrostatic potential (ESP) surface and frontier molecular orbital (FMO) distributions are shown in Figure 2. The ESP map (Figure 2a) reveals a highly polarized charge distribution, characterized by a pronounced negative potential localized on the carbonyl oxygen, identifying this group as the primary hydrogen-bond acceptor and electron-rich interaction site. In contrast, most of the molecular surface exhibits positive ESP values, reflecting the bicyclic scaffold and protonated tertiary nitrogen, and suggesting favorable electrostatic attraction toward negatively charged residues such as Asp and Glu in protein binding pockets. This clear separation of negative and positive regions indicates an amphiphilic electrostatic profile that can guide ligand orientation within enzyme active sites.

FMO analysis further supports this interpretation. The HOMO surface (Figure 2b) is mainly localized over the fused bicyclic ring system and adjacent C–N region, indicating that these domains constitute the principal electron-donating and π-interaction regions of the molecule. In contrast, the LUMO surface (Figure 2c) is concentrated around the carbonyl moiety and neighboring atoms, highlighting these positions as the most electron-deficient and preferred sites for electron acceptance. The spatial separation of HOMO- and LUMO-dominated regions suggests an internal charge-transfer character, consistent with the polarized ESP distribution.

Quantitative ESP analysis mapped onto the electron-density isosurface confirms this electronic asymmetry, with electrostatic potential values spanning from −48.63 to +17.62 kcal/mol and the strongest negative potential localized on the carbonyl oxygen. Together, the ESP metrics, molecular surface properties, and FMO energetics define a complementary electronic profile in which the ring system functions as an electron donor and the carbonyl group acts as a dominant electron acceptor. This electronic organization provides a clear rationale for the observed binding orientation and interaction behavior of N-methylcytisine within the active sites of both AChE and GSK-3β.

### 3.3 Molecular docking

**Figure 3:**
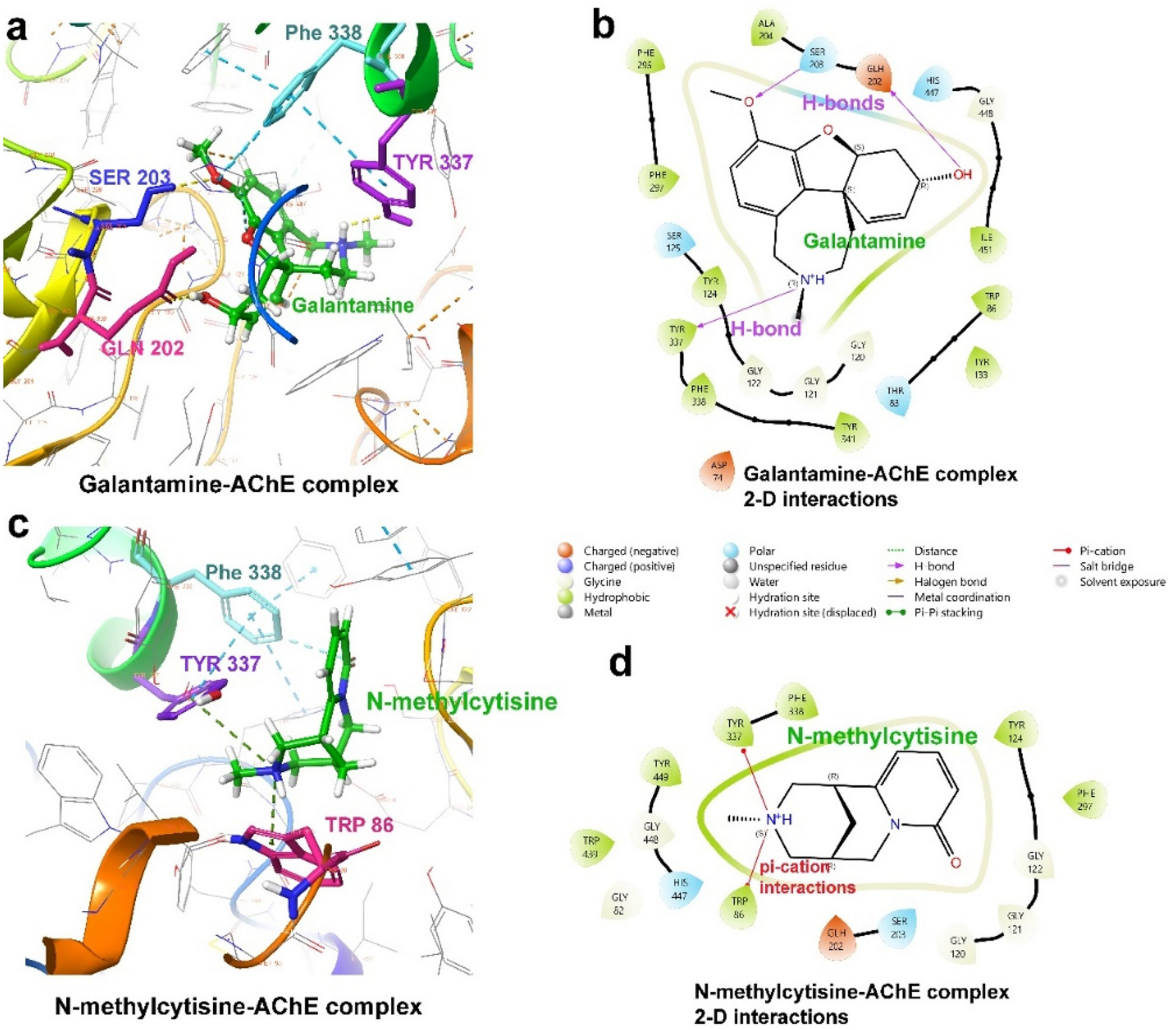
Docking poses of Galantamine and N-methylcytisine in AChE. Panels (a) and (b) show the 3-D binding pose and corresponding 2-D interaction profile of Galantamine within the AChE binding pocket. Panels (c) and (d) show the corresponding binding pose and interaction profile of N-methylcytisine, including interactions with residues surrounding the ligand within the binding site.

Molecular docking analyses were conducted to evaluate the binding of N-methylcytisine to AChE and GSK-3β, using galantamine and staurosporine as reference ligands, respectively. As shown in Figure 3a and b, galantamine binds along the AChE catalytic gorge, forming an extensive hydrogen-bonding network with key residues such as Ser203 and Gln202, together with strong aromatic and π-stacking interactions involving Tyr337 and Phe338. These interactions account for its favorable docking score (−10.8 kcal/mol) and reproduce its known crystallographic binding mode. In comparison, Figure 3c and d demonstrate that N-methylcytisine occupies a similar region of the gorge, where its protonated nitrogen establishes a prominent cation-π interaction with Trp86 and its bicyclic scaffold is stabilized by aromatic contacts with Tyr337 and Phe338. Although fewer classical hydrogen bonds are observed, these interactions largely compensate, resulting in a comparable docking score (−9.6 kcal/mol) and indicating that N-methylcytisine can mimic key recognition features of galantamine within AChE.

**Figure 4:**
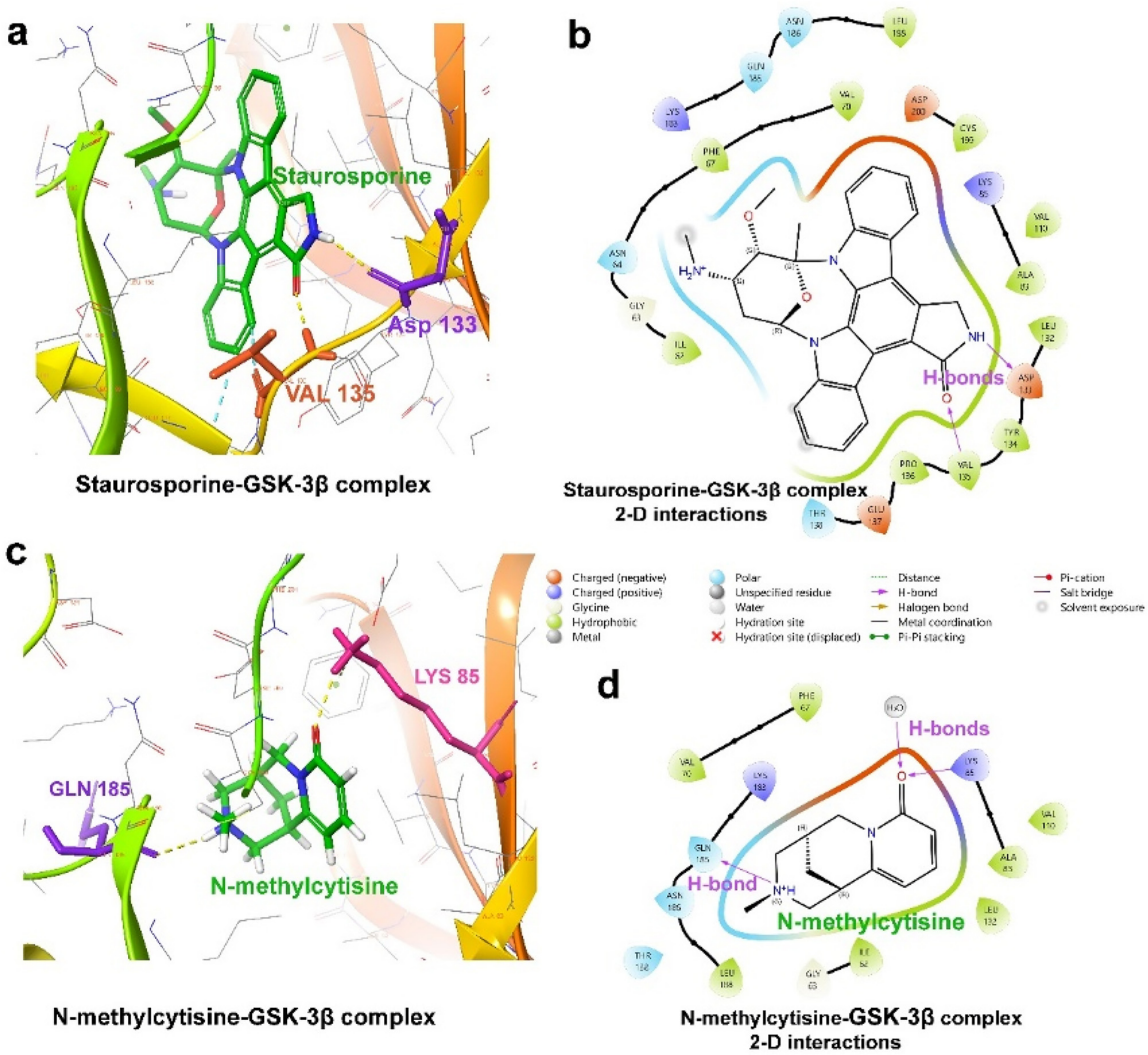
Docking poses of Staurosporine and N-methylcytisine in GSK-3β. Panels (a) and (b) show the 3-D binding pose and corresponding 2-D interaction profile of Staurosporine, highlighting interactions within the ATP-binding pocket and hinge region. Panels (c) and (d) show the corresponding binding pose and interaction profile of N-methylcytisine, which occupies a more compact region of the binding pocket and forms a smaller hydrogen-bonding network.

Docking results for GSK-3β are summarized in Figure 4. In the staurosporine–GSK-3β complex (Figure 4a and b), the ligand binds deeply within the ATP-binding cleft, forming multiple hinge-region hydrogen bonds, particularly with residues such as Asp133 and Val135, along with extensive hydrophobic interactions. This dense interaction network explains its strong docking score (−8.5 kcal/mol) and validates its role as a reference kinase inhibitor. By contrast, Figure 4c and d shows that N-methylcytisine adopts a more modest binding mode within the GSK-3β active site, forming fewer stabilizing hydrogen bonds, notably with Gln185, and limited additional polar contacts. The smaller, rigid scaffold of N-methylcytisine restricts its ability to fully occupy the ATP pocket, leading to reduced interaction density and a lower docking score (−5.3 kcal/mol). Nevertheless, the observed poses indicate that N-methylcytisine can stably engage the GSK-3β binding cleft, albeit less extensively than the larger staurosporine molecule.

In contrast, N-methylcytisine is a considerably smaller bicyclic ligand, and therefore its reduced number of contacts does not necessarily reflect poor intrinsic affinity, but rather the expected limitation in surface area available for protein engagement. Despite its smaller size, N-methylcytisine successfully anchors within the GSK-3β cleft, forming key polar interactions (e.g., with Gln185 and Lys85) and maintaining a stable pose with GSK-3β. Thus, the docking results support the notion that N-methylcytisine achieves efficient binding relative to its compact scaffold, even though it does not recreate the extensive hinge-binding motifs characteristic of larger inhibitors like staurosporine. The results are summarized in Table 2.

**Table 2:** Molecular docking results, docking scores, and key interaction residues for the molecules Galantamine, Staurosporine, and N-methylcytisine with respect to AChE and GSK-3β.

Molecule	Target	Docking score (kcal/mol)	Key interaction residues
Galantamine	AChE	−10.8	Ser203, Gln202, Tyr337, Phe338, Phe295/297 (Hydrogen bonds and hydrophobic interactions)
N-methylcytisine	AChE	−9.6	Trp86 (cation–π interaction), Tyr337, Phe338 (Hydrophobic/π interactions)
Staurosporine	GSK-3β	−8.5	Asp133, Val135, Gln185, Lys85, Hinge-region residues (Multiple hydrogen bonds, hydrophobic contacts)
N-methylcytisine	GSK-3β	−5.3	Gln185, Lys85 (Hydrogen bonds, polar interactions)

### 3.4 Molecular dynamics (MD) simulations

**Figure 5:**
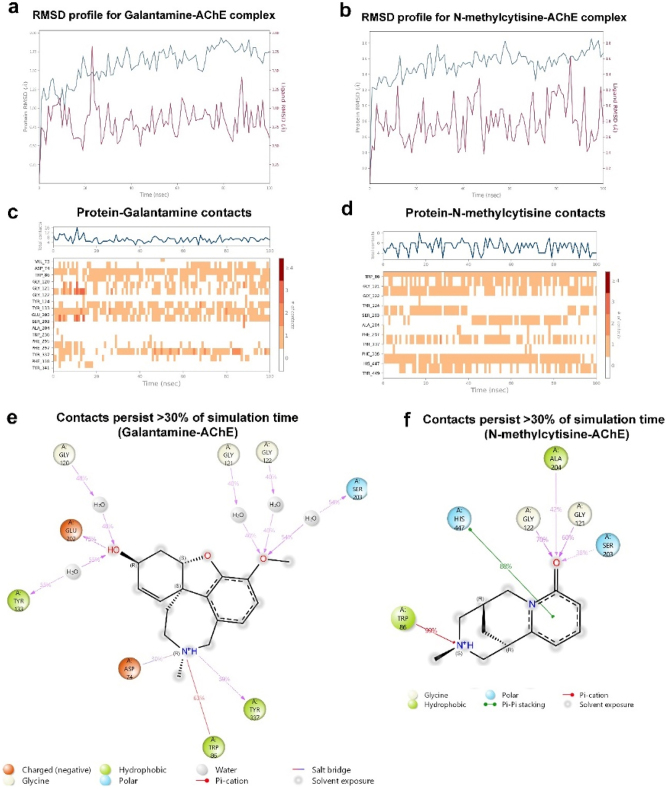
MD simulation analysis of AChE complexes with Galantamine and N-methylcytisine over 100 ns. (a–b) Protein and ligand RMSD profiles showing initial structural adjustment followed by relatively stable protein trajectories, while the ligands exhibit larger short-term fluctuations without progressive displacement from the binding site. (c–d) Protein–ligand contact timelines showing recurring interactions with binding-site residues throughout the simulations. (e–f) Two-dimensional interaction maps summarizing contacts persisting for more than 30 % of the trajectory, demonstrating persistent interaction networks for both Galantamine and N-methylcytisine within AChE.

The molecular dynamics (MD) results for the AChE complexes with galantamine and N-methylcytisine are summarized in Figure 5. The RMSD profiles shown in Figure 5a and b indicate that both AChE–galantamine and AChE–N-methylcytisine complexes remain structurally stable over the 100 ns simulation. Galantamine exhibits slightly lower RMSD fluctuations (Figure 5a), consistent with its well-established binding mode as a clinically approved AChE inhibitor. N-methylcytisine reaches equilibrium after a short adjustment phase and maintains a stable trajectory (Figure 5b), demonstrating its sustained accommodation within the AChE catalytic gorge.

Protein–ligand contact analyses over the simulation time are presented in Figure 5c and d. Galantamine (Figure 5c) preserves persistent hydrogen bonds with SER203 and GLU202, together with hydrophobic interactions involving TYR337 and PHE338, reflecting its canonical interaction pattern. N-methylcytisine (Figure 5d) also maintains stable contacts with key residues, including TRP86, TYR337, GLY121, and SER203, despite its smaller scaffold, and exhibits adaptive yet continuous engagement of the active site. The fraction of interactions persisting for more than 30 % of the simulation is illustrated in Figure 5e and f. Galantamine (Figure 5e) forms multiple long-lived hydrogen bonds and aromatic contacts, consistent with its stronger docking score (−10.8 kcal/mol). In comparison, N-methylcytisine (Figure 5f) shows fewer but still significant persistent interactions, notably with TYR124, GLY121, HIS447, and SER203, supported by occasional water-mediated bridges. Overall, these MD results confirm that N-methylcytisine remains stably bound within the AChE active site and adopts a viable interaction pattern, in agreement with its favorable docking score (−9.6 kcal/mol).

**Figure 6:**
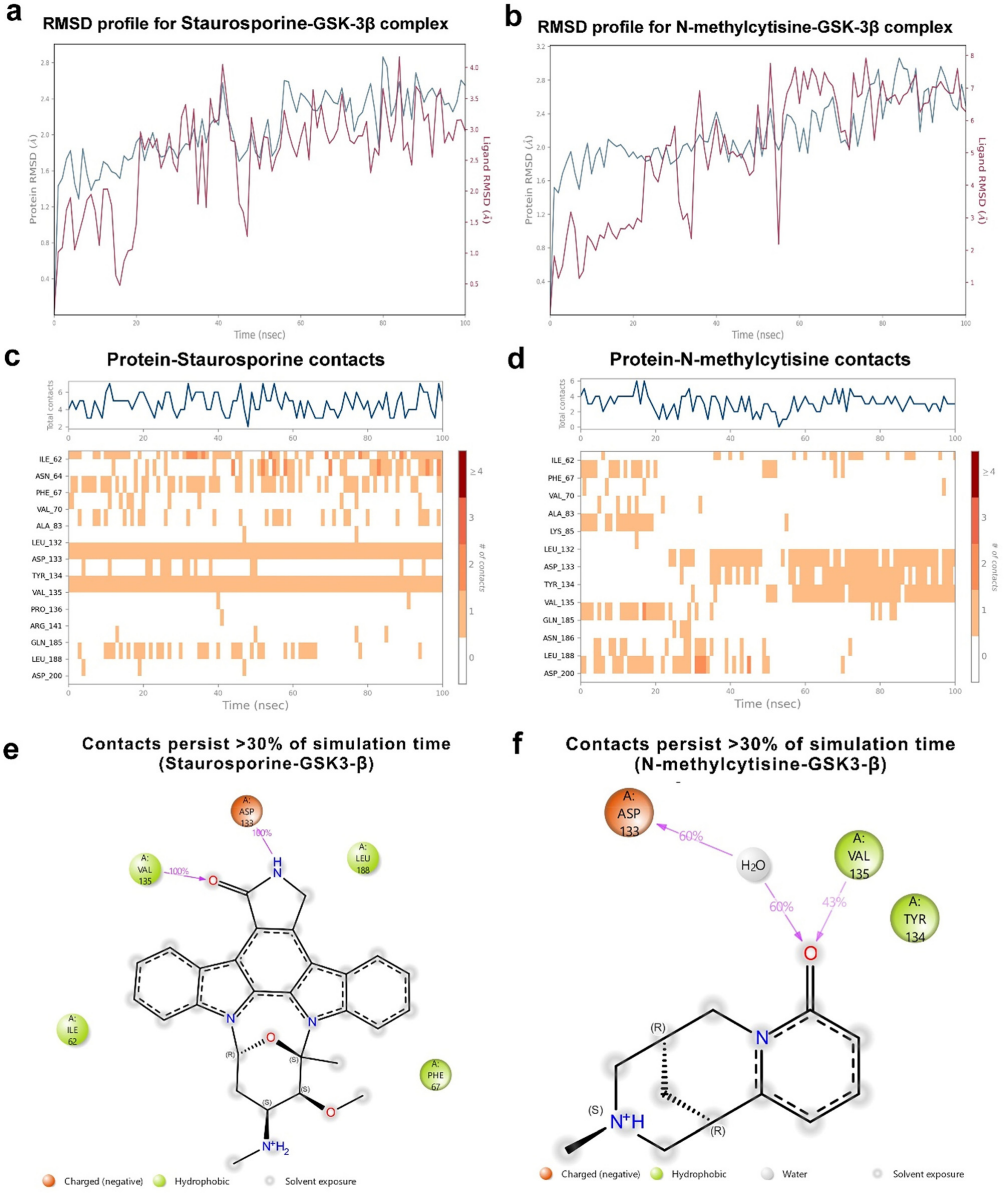
MD simulation analysis of GSK-3β complexes with Staurosporine and N-methylcytisine over 100 ns. (a–b) Protein and ligand RMSD profiles showing structural adjustment followed by fluctuating trajectories, with transient ligand deviations but no sustained displacement from the binding site. (c–d) Protein–ligand contact timelines illustrating dynamic but recurring interactions throughout the simulations. (e–f) Two-dimensional interaction maps summarizing contacts persisting for more than 30 % of the trajectory. Staurosporine maintains a broader network of persistent contacts, including interactions in the hinge region, whereas N-methylcytisine displays a more localized but persistent interaction pattern within the GSK-3β binding pocket.

The molecular dynamics (MD) analysis of GSK-3β complexes with staurosporine and N-methylcytisine is summarized in Figure 6. The RMSD profiles shown in Figure 6a and b indicate that the staurosporine–GSK-3β complex exhibits highly stable behavior throughout the 100 ns simulation, consistent with its classical hinge-binding, ATP-competitive inhibition mode. In contrast, N-methylcytisine displays moderately higher RMSD fluctuations (Figure 6b), reflecting its smaller size and reduced steric complementarity to the ATP-binding pocket; nevertheless, the ligand remains consistently bound and does not dissociate during the simulation.

Protein–ligand contact timelines are presented in Figure 6c and d. Staurosporine (Figure 6c) maintains dense and persistent interactions with key hinge-region residues (ASP133, VAL135, TYR134), activation-loop residues, and extensive hydrophobic surfaces, explaining its strong docking score (−8.5 kcal/mol) and known pan-kinase affinity. N-methylcytisine (Figure 6d) forms a simpler yet stable interaction network, with prominent contacts involving VAL70, ALA83, LYS85, ASN64, and ASP133, and retains at least one stabilizing interaction at most time points. The fraction of interactions persisting for more than 30 % of the simulation is shown in Figure 6e and f. Staurosporine (Figure 6e) exhibits multiple long-lived hydrogen bonds and hydrophobic contacts that anchor it deeply within the ATP pocket. Although N-methylcytisine lacks the polycyclic scaffold required for extensive hinge coverage, it still shows persistent interactions with key residues such as ASP133, VAL135, and TYR134, supported by water-mediated hydrogen bonds (Figure 6f). Overall, these MD results confirm that N-methylcytisine can adopt a flexible yet stable binding mode within the GSK-3β active site, supporting its docking results (−5.3 kcal/mol) and reinforcing experimental indications of its promising GSK-3β inhibitory potential.

### 3.5 MM-GBSA free energy calculations

The MM-GBSA calculations provide a refined evaluation of binding energetics by accounting for solvation and enthalpic contributions beyond docking scores. The averaged MM-GBSA results for all ligand–protein complexes are summarized in Table 3. For the AChE systems, galantamine (−70.6 ± 5.05 kcal/mol) and N-methylcytisine (−70.2 ± 6.14 kcal/mol) exhibit nearly identical binding free energies, indicating comparable stabilization of the AChE–ligand complexes. This energetic similarity supports the docking and MD findings, confirming that N-methylcytisine fits well within the AChE catalytic gorge and maintains persistent interactions with key residues, consistent with meaningful inhibitory potential. In contrast, a clearer distinction is observed for the GSK-3β complexes. Staurosporine shows a strong binding free energy (−82.6 ± 8.16 kcal/mol), reflecting its extensive interaction network and high affinity within the ATP-binding pocket. N-methylcytisine displays a weaker MM-GBSA value (−50.3 ± 6.57 kcal/mol), which can be attributed to its smaller size and reduced contact surface. Nevertheless, this energy range is typical for small natural scaffolds that engage kinases through partial pocket and hinge-region interactions. When considered together with its stable MD behavior and favorable ADMET profile, the MM-GBSA results suggest that N-methylcytisine acts as a moderate yet viable GSK-3β modulator rather than a strong ATP-competitive inhibitor. Overall, the MM-GBSA analysis reinforces two key conclusions: N-methylcytisine closely matches galantamine in AChE binding energetics, and although weaker than staurosporine, it maintains an energetically relevant and stable interaction with GSK-3β. These findings further support N-methylcytisine as a promising dual-target natural compound relevant to neurodegenerative disease mechanisms.

**Table 3:** MM-GBSA calculations were obtained by averaging over snapshots obtained from MD simulations.

Ligand-protein complex	MM-GBSA (kcal/mol) ± St.dev.
Galantamine-AChE	−70.6 ± 5.05
N-methylcytisine-AChE	−70.2 ± 6.14
Staurosporine-GSK-3β	−82.6 ± 8.16
N-methylcytisine-GSK-3β	−50.3 ± 6.57

### 3.6 ADMET analysis

The ADMET analysis highlights clear differences in the pharmacokinetic and safety profiles of galantamine, staurosporine, and N-methylcytisine, as summarized in Table 4. Galantamine exhibits a well-optimized CNS drug profile, with moderate lipophilicity (logP 1.2), low TPSA (41.9 Å^2^), confirmed BBB permeability, high predicted intestinal absorption (>85 %), and a non-mutagenic AMES outcome, supporting its established suitability for long-term neurodegenerative therapy. N-methylcytisine shows a similarly favorable, and in some aspects superior, ADMET pattern. Its low molecular weight (206 g/mol), very low TPSA (24.9 Å^2^), and low logP (−0.2) translate into excellent oral absorption, strong BBB permeability, and reduced risk of nonspecific toxicity. Importantly, N-methylcytisine meets all major drug-likeness criteria and is predicted to be non-mutagenic, underscoring its promise as a small, brain-accessible natural scaffold for neuroprotective applications. In contrast, staurosporine, despite its potent kinase inhibitory activity, displays several limitations for neurodegenerative drug development. Its higher molecular weight (466 g/mol), elevated lipophilicity, and larger TPSA approach or violate key drug-likeness thresholds, and its known kinase promiscuity and systemic toxicity make it unsuitable for chronic CNS indications. Consequently, staurosporine’s ADMET profile supports its role as a reference inhibitor rather than a viable therapeutic lead. Overall, the ADMET comparison reinforces that galantamine and N-methylcytisine possess the most appropriate profiles for neurodegenerative drug discovery, with N-methylcytisine emerging as a particularly attractive natural, CNS-directed candidate warranting further investigation.

**Table 4:** ADMET analysis for active molecules.

Parameter	Galantamine	Staurosporine	N-methylcytisine	Reference
Molecular weight (g/mol)	287.35	466.53	206.28	PubChem CID: 9651, 44259, 670971
logP (consensus)	1.2	2.9	−0.2	SwissADME
H-bond donors (HBD)	1	1	1	PubChem
H-bond acceptors (HBA)	4	6	2	PubChem
Topological polar surface area (TPSA, Å^2^)	41.9	84.0	24.9	SwissADME
Human intestinal absorption (HIA, %)	High (>85 %)	High (>90 %)	High (>90 %)	ADMETlab 2.0
Blood–brain barrier (BBB) permeability	Yes (penetrant)	Yes (penetrant)	Yes (penetrant)	SwissADME/literature
AMES mutagenicity	Non-mutagenic	Non-mutagenic	Non-mutagenic	ADMETlab 2.0
Lipinski rule of 5	Passes	Violates (MW > 500, high lipophilicity)	Passes	SwissADME

## 4 Discussion

Alzheimer’s disease (AD) is increasingly recognized as a multifactorial disorder involving cholinergic dysfunction, tau hyperphosphorylation, oxidative stress, and neuroinflammation. Consequently, multitarget-directed ligands (MTDLs), capable of simultaneously modulating multiple pathological pathways, have emerged as an attractive therapeutic strategy over conventional single-target agents. Within this context, the present study demonstrates that N-methylcytisine possesses dual inhibitory activity against both AChE and GSK-3β, two key enzymes involved in the progression of AD. Although its inhibitory potency was lower than that of the corresponding reference inhibitors, moderate activity against both enzymes is noteworthy because natural products frequently exhibit multitarget pharmacological effects through complementary mechanisms rather than the high potency characteristic of synthetic single-target drugs. Previous studies have consistently reported that cytisine and several of its naturally occurring derivatives possess weak or negligible cholinesterase inhibitory activity [28], 29]. In contrast, the present findings indicate that N-methylcytisine exhibits appreciable inhibition of AChE, suggesting that N-methyl substitution may favorably modify the interaction of the quinolizidine scaffold with the catalytic site of the enzyme. Similar structure-dependent modulation of cholinesterase inhibition has previously been described among quinolizidine alkaloids, where relatively minor structural modifications substantially influenced biological activity [28]. Therefore, the enhanced cholinergic inhibition observed in the present study may represent an important pharmacological consequence of N-methylation rather than a general property of the cytisine scaffold. Likewise, N-methylcytisine demonstrated moderate inhibitory activity against GSK-3β, which is comparable to the micromolar inhibitory activities reported for several naturally occurring kinase modulators, including quercetin, kaempferol, berberine, harmine, curcumin, and resveratrol [5], [6], [7], [8]. Importantly, enzyme kinetic analysis revealed that N-methylcytisine acts as a mixed-type inhibitor. Unlike purely ATP-competitive inhibitors, mixed-type inhibitors may interact with multiple regions of the enzyme, thereby modulating kinase activity through more than one binding mechanism. Such a mode of inhibition is particularly relevant because GSK-3β undergoes conformational changes associated with tau phosphorylation, amyloid precursor protein processing, and neuronal stress signaling [3]. Consequently, compounds capable of modulating different functional states of GSK-3β may offer broader therapeutic benefits than inhibitors acting exclusively through ATP competition. Similar inhibitory mechanisms have also been proposed for several naturally occurring GSK-3β modulators [7].

To further understand the molecular basis of the experimentally observed enzyme inhibition, molecular docking and molecular dynamics simulations were performed as complementary computational approaches. The reference inhibitors, staurosporine and galantamine, exhibited favorable binding characteristics consistent with their well-established inhibitory mechanisms, thereby validating the computational protocol employed in this study. Compared with these reference compounds, N-methylcytisine displayed lower predicted binding affinity but maintained a stable binding orientation within the active sites of both GSK-3β and AChE throughout the molecular dynamics simulations. These computational observations are consistent with the moderate inhibitory activity observed experimentally and provide structural insight into the possible molecular interactions responsible for enzyme inhibition. However, the docking, molecular dynamics, DFT, and ADMET analyses should be interpreted as supportive evidence rather than definitive proof of biological activity.

The predicted ADMET profile further suggested that N-methylcytisine possesses favorable physicochemical properties, including predicted blood-brain barrier permeability and the absence of major predicted mutagenicity alerts, characteristics that are desirable for central nervous system drug candidates. Nevertheless, these predictions require experimental validation through pharmacokinetic, toxicological, and cellular investigations before therapeutic applicability can be established. Accordingly, the computational analyses presented herein primarily serve to complement the *in vitro* enzyme inhibition data by providing mechanistic insights into ligand-enzyme interactions rather than demonstrating clinical efficacy.

Collectively, the present findings suggest that N-methylation distinguishes N-methylcytisine from its parent alkaloid, cytisine, by conferring a unique dual inhibitory profile against two therapeutically relevant Alzheimer’s disease targets. This observation places N-methylcytisine conceptually alongside naturally occurring multitarget compounds [5], [6], [7], [8]. To the best of our knowledge, this is the first study demonstrating dual inhibitory activity of a cytisine-derived alkaloid against both AChE and GSK-3β. Although these findings provide a promising basis for future investigation, additional studies employing neuronal cell models, pharmacokinetic evaluation, and *in vivo* models of Alzheimer’s disease are necessary to determine whether the observed enzymatic inhibition translates into meaningful neuroprotective efficacy.

## 5 Conclusions

The present study demonstrates that N-methylcytisine exhibits dual inhibitory activity against AChE and GSK-3β, supported by complementary molecular docking, molecular dynamics, MM-GBSA, and ADMET analyses that provide mechanistic insight into its interactions with both targets. Although its inhibitory potency was lower than that of the corresponding reference inhibitors, N-methylcytisine displayed a mixed-type inhibitory mechanism against GSK-3β and favorable predicted pharmacokinetic properties, supporting its potential as a multitarget lead compound. Collectively, these findings suggest that N-methylcytisine represents a promising quinolizidine alkaloid for further investigation in Alzheimer’s disease. Nevertheless, additional cellular, pharmacokinetic, toxicological, and *in vivo* studies are required to validate its predicted properties and to determine its therapeutic potential.
